# A Low-Cost, Easy-to-Assemble Device to Prevent Infant Hyperthermia under Conditions of High Thermal Stress

**DOI:** 10.3390/ijerph182413382

**Published:** 2021-12-19

**Authors:** Ramon Farré, Miguel A. Rodríguez-Lázaro, Anh Tuan Dinh-Xuan, Martí Pons-Odena, Daniel Navajas, David Gozal

**Affiliations:** 1 Unitat de Biofísica i Bioenginyeria, Facultat de Medicina i Ciències de la Salut, Universitat de Barcelona, 08036 Barcelona, Spain; m.rodriguez@ub.edu (M.A.R.-L.); dnavajas@ub.edu (D.N.); 2 CIBER de Enfermedades Respiratorias, 28029 Madrid, Spain; 3 Institut Investigacions Biomèdiques August Pi Sunyer, 08036 Barcelona, Spain; 4 Service de Physiologie-Explorations Fonctionnelles, Hôpital Cochin, Assistance Publique-Hôpitaux de Paris (AP-HP), 75014 Paris, France; anh-tuan.dinh-xuan@aphp.fr; 5 Immune and Respiratory Dysfunction Research Group, Institut de Recerca Sant Joan de Déu, Pediatric Intensive Care and Intermediate Care Department, Sant Joan de Déu University Hospital, Universitat de Barcelona, 08950 Esplugues de Llobregat, Spain; mpons@sjdhospitalbarcelona.org; 6 The Institute for Bioengineering of Catalonia (IBEC), The Barcelona Institute of Science and Technology (BIST), 08028 Barcelona, Spain; 7 Department of Child Health, The University of Missouri School of Medicine, Columbia, MO 65201, USA; gozald@health.missouri.edu

**Keywords:** hyperthermia, heatwave, heat shock, heat index, low-middle income countries, air conditioning, negative heat transfer, high ambient temperature, low-cost refrigeration

## Abstract

High ambient temperature and humidity greatly increase the risk of hyperthermia and mortality, particularly in infants, who are especially prone to dehydration. World areas at high risk of heat stress include many of the low- and middle-income countries (LMICs) where most of their inhabitants have no access to air conditioning. This study aimed to design, evaluate, and test a novel low-cost and easy-to-assemble device aimed at preventing the risk of infant hyperthermia in LMICs. The device is based on optimizing negative heat transfer from a small amount of ice and transferring it directly to the infant by airflow of refrigerated air. As a proof of concept, a device was assembled mainly using recycled materials, and its performance was assessed under laboratory-controlled conditions in a climatic chamber mimicking realistic stress conditions of high temperature and humidity. The device, which can be assembled by any layperson using easily available materials, provided sufficient refrigerating capacity for several hours from just 1–2 kg of ice obtained from a domestic freezer. Thus, application of this novel device may serve to attenuate the adverse effects of heat stress in infants, particularly in the context of the evolving climatic change trends.

## 1. Introduction

Body temperature regulation requires a balance between the heat produced by metabolic biochemical reactions and the heat interchanged with the environment. Heat transfer occurs by conduction, convection and radiation, and depends on the difference between body (37 °C) and ambient (*T_amb_*) temperatures. Heat dissipation by water evaporation (through breathing and mainly transpiration) depends on the partial pressure of water vapor in the air and hence on ambient relative humidity (*RH_amb_*) [1,2,3]. In temperate climates, *T_amb_* and *RH_amb_* are usually below 37 °C and 100%, respectively, and therefore all four of these of mechanisms of heat dissipation effectively contribute to body heat loss, thereby readily achieving normothermia. However, in hot climates, normothermia can be compromised because heat dissipation by conduction, convection and radiation is reduced as *T_amb_* approaches 37 °C, and in cases where *T_amb_* > 37 °C, heat flow direction is reversed, and the body is heated by the environment. Therefore, in hot climates, water evaporation is the principal mechanism, and, when *T_amb_* > 37 °C, the only mechanism, for heat dissipation. If *RH_amb_* is sufficiently low, normothermia may be preserved because increased transpiration can generate the required heat losses. However, in addition to high *T_amb_*, another condition that potentially compromises body temperature regulation is high *RH_amb_* because heat dissipation by water evaporation is reduced as *RH_amb_* increases. When *RH_amb_* = 100%, evaporation is an ineffectual mechanism for heat dissipation. Therefore, both high *T_amb_* and *RH_amb_* increase transpiration (and respiratory) rate with the risk of excessive body water loss, particularly in infants, because they are prone to dehydration even when healthy [4]. Remarkably, the most critical situation arises when *T_amb_* exceeds 37 °C and *RH_amb_* approaches 100%, because the body may be unable to dissipate enough heat by evaporation, leading to subsequent dehydration, increase in body core temperature, heatstroke, coma, and ultimately death.

Accordingly, mortality risk under thermal stress can be better explained by considering both *T_amb_* and *RH_amb_* [5,6,7,8]. To this end, a variable such as the Heat Index (*HI*) was constructed to provide a single value that integrates the effects of *T_amb_* and *RH_amb_* to estimate thermal risk [9]. Although it is an empiric variable that does not perfectly captures the whole process of body heat interchange and temperature homeostasis, *HI* provides a useful scale (‘caution’, ‘extreme caution’, ‘danger’, and ‘extreme danger’) to quantify the risk under high temperature/humidity stress conditions [10]. The latter occur, at least during some months of the year, in low- and middle-income countries (LMICs), many of which are in tropical and equatorial regions (e.g., Amazon basin, north and central Africa, Middle East, Indian subcontinent, and Southeast Asia), and are inhabited by an important percentage of the world population [11,12]. A documented consequence of augmented thermal stress by high *T_amb_* and *RH_amb_* is an increase in pediatric mortality in developed countries [13,14,15] and particularly in LMICs [16,17,18]. Unfortunately, the current situation of health thermal risk worldwide is progressively worsening because of global warming and associated climate change [19,20,21,22].

The most obvious means of reducing the health risks caused by high *HI* is the use of air conditioning to establish safe *T_amb_* and *RH_amb_* in residential buildings. Air conditioning is widely used in developed countries, but in LMICs it is accessible only to a very small minority of the population. Indeed, according to 2015 data from the International Energy Agency, air conditioning in households was available to just 8% of the 2.8 billion people living in the hottest regions of the world [23]. Considering that the socio-economic situation of most inhabitants in LMICs has not significantly changed in recent years, nor is it expected to improve in the medium term, actions to minimize the thermal risk in infants either in LMIC homes or hospital wards that have no air conditioning are urgently required. Under high *HI* conditions, it would be useful to enhance body heat losses by streaming a small flow of refrigerated air onto the surface of the infant’s body. Given that such a refrigerated air source is not readily available among most potential users in LMICs, we designed and tested a novel, low-cost and easy-to-assemble device that should help reduce the thermal risk in infants. The rationale of the approach is to optimize negative heat transfer from a modest amount of ice to the infant body, such that a common domestic freezer is de facto the only item required. 

## 2. Materials and Methods

### 2.1. Theoretical Analysis 

**Figure 1:**
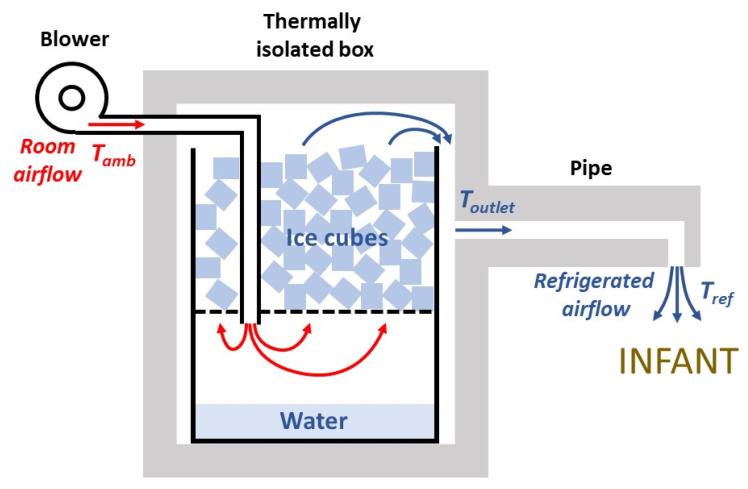
Generator of refrigerated air. A container has two compartments separated by a hollowed surface: ice cubes are placed in the top compartment and water resulting from ice thawing drops to the bottom compartment. The container is enclosed in a box made with thermally isolating walls. A room airflow at ambient temperature *T_amb_* is generated by a blower, enters the lower compartment, and is refrigerated as it passes through the ice cubes. The cooled air leaves the chamber at temperature *T_outlet_* and enters a thermally isolated pipe conducting the airflow to the infant. Given heat dissipation through the pipe wall, the airflow circulating through the pipe is heated to *T_ref_*, which is the temperature of the refrigerated air leaving the device and directed onto the infant. Accordingly, the airflow first experiences a cooling process (see Section 2.1.1) as it passes through the ice cubes and a subsequent heating process inside the pipe (see Section 2.1.2).

Figure 1 shows a diagram of the designed generator of refrigerated air. An airflow of room air is introduced by a blower, and is refrigerated by passing it through ice cubes, then leaving the setting through an outlet pipe placed on top of the infant. To minimize wasting of the ice refrigeration capacity, the setting is enclosed in a thermally isolated box. First, a theoretical model was delineated to assess whether the assembly in Figure 1 can feasibly provide sufficient refrigeration power. The negative heat transfer from ice thawing is spent on three processes: cooling of the airflow, heat transfer through the chamber walls, and condensation of water vapor from the airflow.

#### 2.1.1. Air Cooling Process in the Chamber 

When the average temperature inside the chamber (*T_chamber_*) (Figure 1) is lower than the ambient temperature (*T_amb_*), the heat transfer rate (*Q’_wall_*) into the chamber through the walls having thickness *d*, total surface *A*, and made of a material with thermal conductivity *K_wall_*, is

*Q’_wall_* = *K_wall_*·*A*·(*T_amb_* − *T_chamber_*)/*d* (1)

The negative heat transfer rate (*Q’_air_*) to an airflow *V’* of density *ρ* (1.2 g·L^−1^) and specific heat capacity *c* (1 J·g^−1^·K^−1^) when its temperature is reduced from *T_amb_* to *T_outlet_* is

*Q’_air_* = *V’*·*ρ*
*c*·(*T_amb_* − *T_outlet_*) (2)

The mass (*m*) of water vapor contained in a volume *V* of air at *T* and *RH* is

*m* = [*Wt_mol_*·*V*·*P_H2O_* (*T*)·*RH*]/[100·*R*·*T*] (3)

where *Wt_mol_* is the water molecular weight (18 g·mol^−1^), *R* is the gas constant (8.31 J·K^−1^·mol^−1^), and *P_H2O_*(*T*) is the saturated water vapor pressure at *T* [24]. When an airflow *V’* at *T_amb_* and *RH_amb_* is refrigerated to *T_outlet_* and is water vapor saturated (*RH_outlet_* = 100%)*,* water vapor will be condensed if the difference between the air content of water vapor before and after refrigeration is positive. In that case, considering that the latent heat of water vaporization is *C_v_* (2424 J·g^−1^), the rate of heat required for water condensation for an airflow *V’,* is

*Q’_con_* = [*C_v_*·*V’*·*Wt_mol_*/*R*]·[*P_H2O_*(*T_amb_*)·*RH_amb_*/(100·*T_amb_*) − *P_H2O_*(*T_outlet_*)/*T_outlet_*] (4)

Hence, the total heat transfer rate to the ice, which causes its thawing, is

*Q’* = *Q’_wall_* + *Q’_air_* + *Q’_con_* (5)

Given the latent heat of ice fusion *C_f_* (334 J·g^−1^), the ice thawing rate R corresponding to a heat transfer *Q’* is

R = *Q’*/*C_f_* (6)

For instance, in the case of very hot and dry desert conditions, such as *T_amb_* = 45 °C, *RH_amb_* = 20%, if the chamber is cubic with lateral length 25 cm (*A* = 0.375 m^2^), *d* = 4 cm, the wall material is expanded polystyrene (*K* = 0.035 W·K^−1^·m^−1^), *T_chamber_* = 5 °C, *T_outlet_* = 15 °C, and airflow is V’ = 0.5 L·s^−1^, then *Q’_wall_* = 13.1 W, *Q’_air_ =* 18.0 W, *Q’_con_* = 0.3 W, and thus *Q’* = 31.4 W). Accordingly, the rate of ice thawing would be R = 0.339 kg·h^−1^, and an initial ice load of 1.5 kg into the chamber would last for 4.4 h. For the same assembly, in the case of typical hot and humid tropical conditions of *T_amb_* = 35 °C, *RH_amb_ =* 60%*,* then *Q’_wall_* = 9.8 W, *Q’_air_ =* 12.0 W, *Q’_con_* =13.2 W, indicating that the heat rate corresponding to water vapor condensation would be considerably increased. As, in this case, *Q’* = 35.1 W, the rate of ice thawing would be R = 0.378 kg·h^−1^, and an initial ice load of 1.5 kg into the chamber would last for 4.0 h. Theoretically, these ice lasting times (4.4 and 4.0 h) would be slightly longer because the negative heat transfer corresponding to heating the ice in the chamber (from ≈−15 °C when obtained from a domestic freezer) is ignored.

#### 2.1.2. Air Heating Process in the Pipe

The airflow leaving the isolated box at *T_outlet_* should be conducted close to the infant by a pipe (Figure 1). Because of heat transfer through the pipe walls, the airflow will be heated to the final temperature of the refrigerated air leaving the device (*T_ref_*). The wall heat transfer in a constant-section pipe of length *L*, a material wall with thermal conductivity *K_pipe_*, external temperature *T_amb_* and mean internal temperature (*T_outlet_* + *T_ref_*)/2 is

*Q’_wall,pipe_* = 2π·*L*·*K_pipe_*·*C*·[*T_amb_* − (*T_outlet_* + *T_ref_*)/2] (7)

where *C* is a geometrical factor that depends on the section geometry of the pipe [25]. Given that the pipe has a constant section, considering a uniform internal pipe temperature equal to (*T_outlet_* + *T_ref_*)/2 is equivalent to accounting for the linear temperature decrease inside the pipe. 

If the pipe is cylindrical with internal and external radii *r_i_* and *r_e_*, respectively,

*C_cyl_* = 1/ln(*r_e_*/*r_i_*) (8)

If the pipe has a square section with internal and external wall widths *w_i_* and *w_e_*, respectively,

*C_square_* = 1/[0.93·ln(0.948·*w_e_*/*w_i_*)] (9)

The heat rate to increase the airflow *V’* in the pipe from *T_outlet_* to *T_ref_* is

*Q’_wall,air_* = *V’·ρ·c*·(*T_ref_* − *T_outlet_*) (10)

Hence, *T_ref_* can be derived by solving *Q’_wall,pipe_* = *Q’_wall,air_*, for *T_ref_* ≤ *T_amb_*:

*T_ref_* = [*T_amb_*·2·*α* − *T_outlet_*·(*α* − 2·*β*)]/[*α* + 2·*β*] (11)

where α = 2*π*·*L*·*K_pipe_*·*C*, and *β* = *V’·ρ·c*.

Thus, adequate thermal isolation of the pipe is very important to minimize the amount of heating of the air flowing through the pipe. For instance, in case that *T_outlet_* = 15 °C, *T_amb_* = 45 °C, *V’* = 0.5 L·s^−1^, and the pipe is a *L =* 20 cm long simple cylindrical plastic (PVC) tube (*K_pipe_* = 0.2 W·K^−1^·m^−1^) with *r_i_* = 1 cm and *r_e_* = 1.3 cm (i.e., 3 mm wall thickness), the final temperature of refrigerated air *T_ref_* would increase to *T_ref_* = 41.6 °C, very close to *T_amb_*. By contrast, if the pipe is *L =* 20 cm long, having a square section, and its walls are made of 4 cm thick expanded polystyrene (*K_pipe_* = 0.035 W·K^−1^·m^−1^) with widths *w_i_* = 1 cm and *w_e_* = 9 cm, then the airflow in the pipe would be scarcely heated from *T_outlet_* because *T_ref_* = 16.1 °C.

Therefore, the theoretical analysis of Equations (1)–(11) indicates that the approach is feasible for reasonable physical dimensions of the setting in the context of stressful thermal conditions.

### 2.2. Practical Implementation

**Figure 2:**
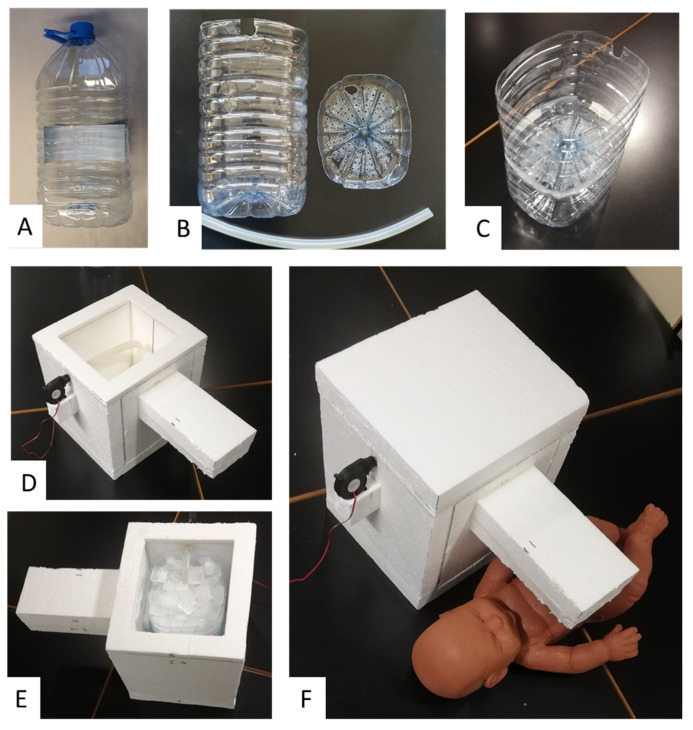
Construction of the simple, low-cost assembly used for the proof-of-concept testing. (**A**) Eight-liter plastic bottle of drinking water. (**B**) Compartment for ice/water made from the lower part of one bottle, piece to separate the ice and water made by cutting and drilling the base of another bottle, and piece of hose. (**C**) Assembled ice/water compartment. (**D**) Isolating chamber (expanded polystyrene) with blower and hose (upper wall open). Glue was used to seal the wall attachments to avoid air leaks. (**E**) Compartment for ice/water placed within the insulating chamber and loaded with ice. (**F**) Setting with closed chamber ready to be used with the testing infant manikin.

Except for a small low-cost blower (DF501512SH FAN, Vordas, Shenzen, China; 12 V; 5 × 5 × 1.5 cm) employed to generate the airflow, we used recycled materials to assemble the device in the diagram of Figure 1. As shown in Figure 2, the ice container was made using two conventional disposable 8 L plastic bottles for drinking water: the upper part of one of the bottles was cut and the bottom of the other bottle was cut and, after making several small holes in it, it was used as the platform to sustain the ice and to allow the melted ice water to drop. The isolating chamber (18 × 22 × 27 cm, internal dimensions) was made using pieces of expanded polystyrene (4 cm width) which is a material usually employed in the building and packaging industries and is therefore widely available (Figure 2). This material was also employed for embedding the external pipe to conduct the refrigerated air to the infant: 20 cm in length, an internal square section of 1 cm per side, and an external rectangular section (4 × 12 cm) (Figure 2). The pipe ended with a vertical, short (3 cm) cylindrical tube having a 12 mm internal diameter (not visible in Figure 2). A 25 cm long piece of hose (1.5 cm in diameter) inside the chamber connected its airflow entrance with the upper part of the water compartment. The length of the tube section inside the water chamber was short enough to avoid it being submersed in water when the ice melts.

### 2.3. Performance Assessment

The setting was tested under laboratory conditions inside a temperature- and humidity-controlled customized climatic chamber (61 × 63 × 83 cm) to mimic real-life thermal stress. Air temperature and relative humidity were continuously measured by a sensor (SHTC3, DollaTek, Hong Kong) and were Arduino-controlled using a heating ventilator (PTC Heater 100 W, 12 V; Fdit, Shenzhen, China) and a humidity nebulizer (Ultra-Neb 2000, Devilbiss, Mannheim, Germany). Airflow *V’* was assessed by measuring pressure at the blower outlet with a transducer (DCXL01DS, Honeywell, Charlotte, NC, USA) and using the previously calibrated pressure–flow relationship of the blower for each driving voltage. The temperature of the refrigerated air (*T_ref_*) at the output of the device and at different distances from the output orifice was measured with a microthermistor (Micro-BetaChip, TE Connectivity, Escafusa, Switzerland). *V’* and *T_ref_* signals were sampled and stored for subsequent analysis. 

An infant manikin (modified from model 2390686, CGTrader, Vilnius, Lithuania) having dimensions corresponding to a ≈6 kg infant was 3D printed (S5, Ultimaker, Utrecht, The Nederland) using polylactic acid (PLA), and finally painted with a layer of brown liquid silicone. Once the refrigerated air source and the infant manikin were at thermal equilibrium within the climatic chamber, 1.7 kg of ice from a domestic freezer was loaded into the device, and the blower was switched on. The temperature at the manikin surface was assessed by a 220 × 160 infrared resolution thermal imaging camera (HT-18, Hti-Xintai, Dongguan City, China).

Measurements for a considerable airflow, *V’* = 0.80 L·s^−1^, were taken for two strenuous conditions, representing very hot dry desert ambient conditions (*T_amb_* = 44 °C, *RH_amb_* = 11%) and hot humid tropical ambient conditions (*T_amb_* = 35 °C, *RH_amb_* = 55%), both labelled as ‘danger’ according to the *HI* (43 and 42 °C, respectively), and for a condition of exceptional stress with *T_amb_* = 36 °C and *RH_amb_* = 70%, labeled as ‘extreme danger’ condition according to the *HI* (52 °C).

## 3. Results

**Figure 3:**
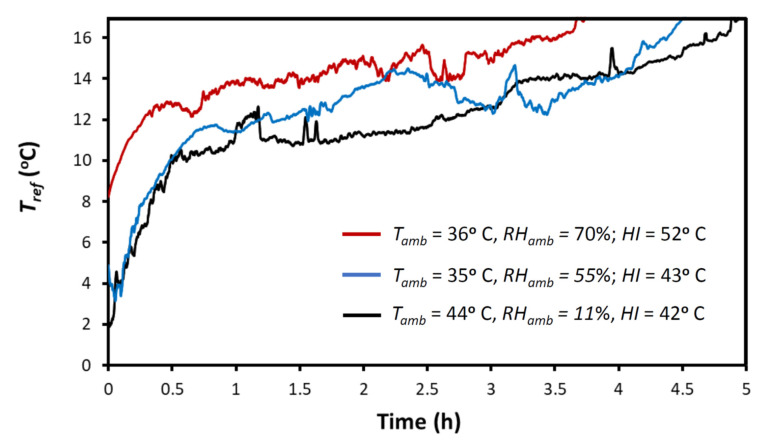
Temperature of the refrigerated airflow leaving the device pipe (*T_ref_*) for different thermally stressing ambient conditions: hot dry desert (black line), hot humid tropical (blue line), and extremely hot and humid tropical (red line). *T_amb_*, *RH_amb_*, and *HI* are ambient temperature, humidity and heat index, respectively. In all these cases, *T_ref_* < 17 °C; hence, the airflow was 20 °C below body temperature (37 °C).

Figure 3 shows the time course of the airflow temperature measured at the pipe orifice (*T_ref_*) for the different thermally stressing conditions tested. For the sake of clarity, only data for *T_ref_* lower than 17 °C are depicted, showing the production of a refrigerated airflow 20 °C below normal body temperature (37 °C), which is most relevant in terms of helping the infant to maintain normothermia. The small fluctuations observed in each *T_ref_* recording were caused by spontaneous ice cube rearrangements as thawing proceeded. However, repeated measurements showed that variations in the time at which *T_ref_* achieved the 17 °C value only varied within a 10 min range (data not shown). As expected, *T_ref_* progressively increased, being <17 °C for 5 h in the example of hot dry desert condition (*HI* = 42 °C), for 4.5 h in the hot humid example of tropical ambient conditions (*HI* = 43 °C), and for 3.75 h in the condition of exceptional stress corresponding to *HI* = 52 °C. Interestingly, the capability of the device to refrigerate the airflow was not limited to the times shown in Figure 3 because, in all cases, at the end of the recording there was still ice remaining within the container. Remarkably, the similar values of airflow temperatures (*T_ref_*) in Figure 3 (at any time all measurements differ by less than ≈5 °C) show that the capacity of the setting for decreasing the airflow temperature scarcely depends on the ambient air conditions (*T_amb_*, *RH_amb_*), although the duration of refrigeration (e.g., for *T_ref_* < 17 °C) considerably decreases with *RH_amb_* due to the energy required for air condensation (as predicted by the theoretical model equations). 

**Figure 4:**
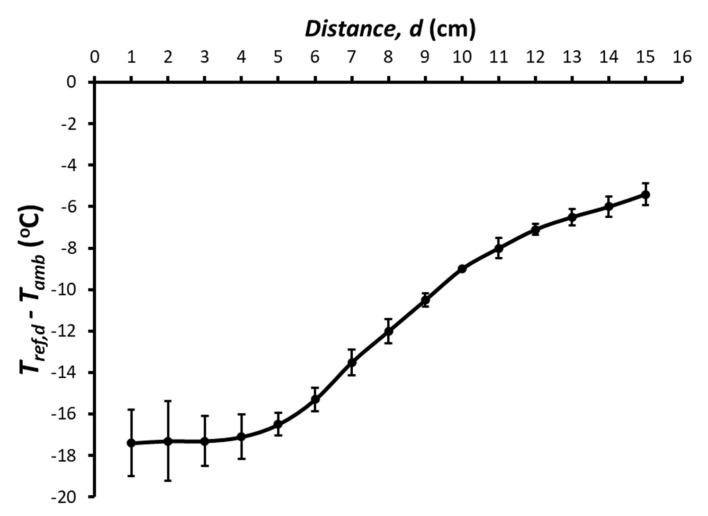
Reduction in the airflow temperature at different distances from the device outlet. The difference between the temperatures of the refrigerated air at a given distance (*d*) (*T_ref,d_*) and the ambient temperature (*T_amb_*) decreased as the distance *d* from the device outlet increased. Data are mean ± standard deviation of three repeated measurements.

Figure 4 shows an example of how the temperature of the airflow (at the vertical below the output orifice of the device) decreases with distance to the outlet orifice. Although the airflow temperature was 17.5 °C below *T_amb_* in the immediate vicinity of the outlet (≈0–5 cm), this temperature drop decreased progressively, being only 5.4 °C below *T_amb_* at a point 15 cm below the airflow outlet. This pattern of temperature dependence on distance and variability is consistent with turbulences and centerline velocity decay in a free jet [26]. Figure 4 shows that, as expected, to optimize the cooling effect of the airflow, the distance from the air outlet to the infant should be reduced as much as possible. This figure also indicates that changing this distance can be a simple means to adapt the setting to the specific cooling needs of the infant.

**Figure 5:**
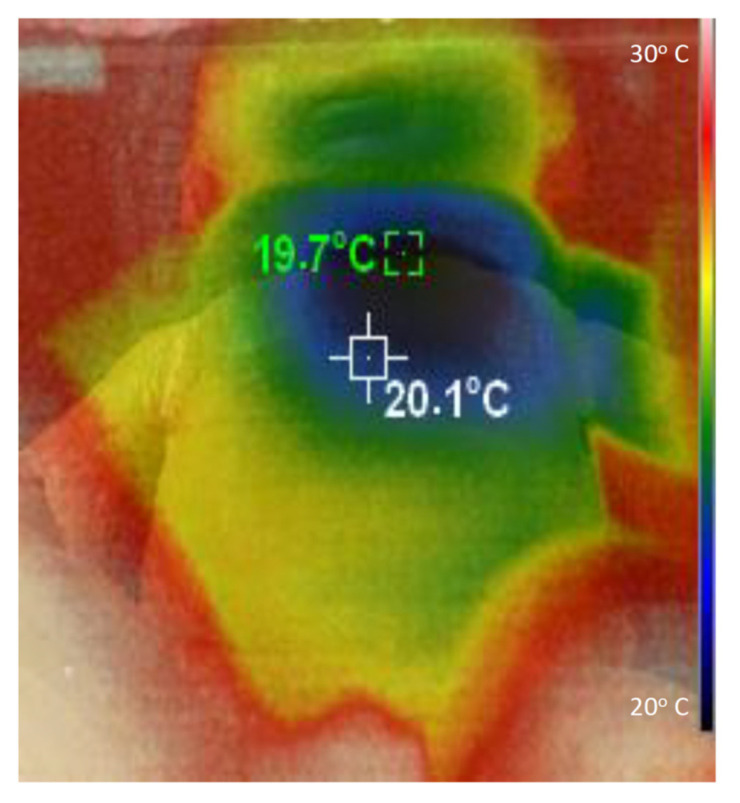
Example showing how the refrigerated airflow from the device is distributed on the manikin surface.

Figure 5 illustrates with an example how the refrigerated air is distributed on the body of the infant. This specific thermal image was taken when the airflow temperature *T_ref_* was 24 °C below *T_amb_*, and the thoracic surface of the manikin was 8 cm below the airflow outlet. It is important to mention that measuring the temperature at the manikin surface simply serves to illustrate how the refrigerated airflow is distributed on the manikin, which has a geometry that realistically mimics that of an infant. The specific values of temperatures in Figure 5 do not represent those that would be found on the skin of an infant because the energetically passive manikin does not dissipate metabolic heat or regulate its temperature.

## 4. Discussion

We here present the rationale, design and feasibility test of a new approach for generating a controlled flow of refrigerated air aimed at preventing dangerous hyperthermia in infants subjected to high temperatures and humidity conditions in LMICs. The theoretical model used to develop the device, as shown in Equations (1)–(11), includes all the energy transfer processes involved and allows any interested designer to dissect the roles played by the different dimensions and materials selected in the device’s construction. As a proof of concept, we built and satisfactorily tested a device that was extremely low cost and easy to fabricate by any layperson equipped with no tools other than a knife and glue (Figure 2). 

The approach in Figure 1 is a practical alternative to other potential means of trying to reduce heat stress in infants when conventional air conditioning is not available. For instance, some proposed approaches at a community level in LMICs are to design buildings, e.g., nurseries, by optimizing architectural structure and materials [27,28]. However, these proposals are expensive, difficult to implement, and do not provide a solution at the individual home level. Regarding home-based approaches, it should be mentioned that the common solution of using a fan for alleviating heat stress is not satisfactory, and in most cases is clearly insufficient. Indeed, if *T_amb_* is slightly below 37 °C, using a fan and thus projecting air at such a high temperature on the infant skin would result in minimal heat loss by convection. Moreover, if *T_amb_* > 37 °C, using a fan would be counterproductive because it would increase heat transfer to the body by convection, further increasing the risk of infant dehydration. Using a cold water bottle is not cost-effective because a high fraction of the negative heat transfer from water is dissipated through the bed and does not reach the infant. Moreover, trying to refrigerate air by placing a pan or bowl with ice cubes in front of a fan has low effectiveness because most ice refrigeration capacity is lost by air dispersion. In fact, the procedure described in Figure 1 is probably the most cost-effective way to achieve optimal negative heat transfer from ice to an infant. 

It is worth noting that the conceptual approach in Figure 1 can be implemented using different configurations. The key point is to allow the airflow to circulate in close contact with the ice surface to reach almost a thermal equilibrium before leaving the chamber. It should be noted that, as the device was designed to achieve the maximum possible negative heat transfer from a modest amount of ice to an infant, optimizing the design according to the model presented in Equations (1)–(11) is crucial to minimize the loss of refrigeration capacity. Specifically, it is important to reduce heat transfer through the walls of the chamber by using a highly isolating material, and by reducing unintended air leaks (using putty in the wall joints may be useful). As a proof of concept, we used expanded polystyrene because, given its low cost and excellent thermal isolation properties, it is widely used in packaging and building industries worldwide, and is therefore available in LMICs either commercially or by recycling from other applications. However, if not available, expanded polystyrene could be replaced by using several layers of common corrugated cardboard recycled from packaging boxes. Indeed, conventional corrugated cardboard has a thermal conductivity ≈2-fold greater than expanded polystyrene [29], indicating that using a cardboard-based chamber wall with 2-fold thickness would provide similar thermal isolation as employing expanded polystyrene. Interestingly, the material of the ice/water container and hose within the chamber (Figure 1) is not relevant because, as they are surrounded by the isolating wall, they play no role in heat exchange. This fact makes it easy to use any new or recycled container and tubing, as shown in the proof-of-concept example (Figure 2). The blower we used was a very low-cost device that is commercialized as a spare component of 3D printers and is available by e-commerce (<EUR 3 retail cost via Amazon and an even lower cost via Alibaba). The blower can be operated by a conventional 12 V source (mains 110/220 V) or by any car/motorbike battery if the conventional power supply is compromised (given its 100 mA consumption at 12 V, any 45 A·h battery from a small car could provide energy for 18 days with 24 h continuous blower operation). 

Some issues deserve to be mentioned regarding the practical application of the proposed device. As shown in Figure 3, the airflow temperature *T_ref_* of the refrigerated airflow leaving the device progressively increased over time. This was expected for a constant airflow *V’* because the amount of ice is progressively reduced. Technically, the cooling capacity of the setting can be easily modulated by incorporating a temperature sensor and a controller to regulate the voltage applied to the blower, but we intentionally avoided any complexity because we aimed to develop an extremely simple and cheap device. Interestingly, the cooling capacity of the setting can be adjusted manually in different manners. One of these is by modifying the distance from the airflow outlet and the infant (Figure 4). Another could be to modify the airflow *V’* of refrigerated air using a power supply that can regulate the voltage provided to the blower (e.g., for the specific setting in Figure 2, when the blower was powered by 9 and 12 V, airflow *V’* was 0.80 and 1.12 L·s^−1^, respectively). In addition, and more simply, airflow can be modified by partially clamping the tube between the blower output and the chamber inlet to reduce *V’*. For this purpose, instead of placing the blower close to the chamber as in Figure 2F, a soft piece of hose could be used to connect the blower and the tube inside the chamber. As the simple device does not include a thermometer, the airflow temperature *T_ref_* cannot be measured, as was done during testing in the laboratory. However, the temperature reaching the infant’s surface, which is the relevant temperature in practice, can be sensed by hand by the infant’s caregiver and modified accordingly. Given its simplicity and low cost, the device was not designed as a precise medical device, but rather as an individual tool to reduce the risk of hyperthermia in infants and young children living in homes and nurseries in LMICs under high thermal stress conditions because of lack of air conditioning.

It is interesting to note that a relatively low flow (e.g., 0.5 L·s^−1^) of refrigerated air has a high potential for dissipating body heat in two ways. When this air is directly applied to the infant’s skin (at 37 °C), he/she will lose heat according to two mechanisms. Theoretically, the refrigerated air (e.g., at 10 °C), when in contact with the skin at 37 °C, will be rapidly heated to this temperature and, hence, according to Equation (2), it will extract heat from the body at a rate of 16.2 W. Moreover, the refrigerated air at 10 °C, although it is saturated with water vapor when leaving contact with the ice, has a relatively low vapor content; hence, when it is heated by contacting the infant’s skin, it can evaporate a considerable amount of water from transpiration, thereby extracting further heat from the body. In fact, as derived from the previous theoretical equations, the refrigerated air in the example has the potential of extracting 25.6 W of heat by evaporation. By adding the potential heat transfer at the skin level, via air heating and water evaporation, 0.5 L·s^−1^ of saturated air at 10 °C air can extract up to 41.8 W from the infant. Remarkably, this refrigerating power is ≈2-fold the typical total resting metabolic rate of an infant weighing 6 kg (19.4 W = 67 kcal·kg^−1^·day^−1^) [30] which, in turn, is greater than his/her heat dissipation rate. Therefore, even if the refrigeration efficiency of the projected airflow on the infant’s skin is considerably below 100%, the device should have enough capacity to prevent infant hyperthermia. However, future studies are required to assess the clinical effectiveness of the approach in infants.

## 5. Conclusions

We designed, implemented, and characterized the effectiveness of a device that can be useful to avoid hyperthermia in infants and young children under severe climatic thermal stress. The device, based on a sound theoretical background, was intentionally devised to be extremely simple and low cost so that it can be implemented in homes in LMICs without air conditioning. In addition to being easy to build, the device is simple to operate because it only requires a modest amount of ice from a conventional freezer to work for several hours. Given that it is based on thermally isolated negative heat transfer from ice, which is achieved by an optimized heat pump technology in a refrigerator, the device is energetically sustainable. From a practical viewpoint, the only consumable that the device requires is ice, which is certainly available, or easy to access, in many homes and nurseries in LMICs that are without air conditioning and experience dangerous heat index conditions. The device, which is highly applicable at present, will undoubtedly become increasingly useful in the future given the increasing trends in average temperatures and heatwaves caused by the current changes in climate [31].

## Data Availability

Data are available from the corresponding author at address: rfarre@ub.edu.
